# The effect of adjuvant imatinib treatment for patients with high-risk gastrointestinal stromal tumor

**DOI:** 10.3389/fonc.2026.1756927

**Published:** 2026-04-27

**Authors:** Jie Zhang, Donghui Gao, Hongming Cui, Jinjin Li, Hang Ao, Xingye Wu

**Affiliations:** Department of Gastrointestinal Surgery, The First Affiliated Hospital of Chongqing Medical University, Chongqing, China

**Keywords:** gastrointestinal stromal tumors, imatinib, plasma concentration, prognosis, surgery

## Abstract

**Background:**

Imatinib mesylate (IM) has changed the management of gastrointestinal stromal tumors (GISTs), especially for adjuvant therapy after radical resection. However, the optimal duration of the adjuvant therapy remains controversial. Therefore, this study evaluated the effect of adjuvant IM duration and plasma drug concentrations on survival outcomes of patients at high risk with GISTs.

**Methods:**

We retrospectively analyzed patients diagnosed with high-risk GIST who underwent radical surgery at the First Affiliated Hospital of Chongqing Medical University between January 2008 and December 2021. Survival outcomes were compared according to adjuvant IM duration (≤3 years vs. >3 years). Plasma trough concentrations (Cmin) of IM were measured in a subset of patients and analyzed in relation to prognosis.

**Results:**

This study included 75 patients (42 males and 33 females), with a median age of 53 (24–79) years. Tumors were most commonly located in the small intestine (44 patients, 58.7%) and stomach (23 patients, 30.7%). Kaplan-Meier curve analysis showed that the 5-year relapse-free survival (RFS) rates were 84% and 74% in the groups with treatment duration >3 years and ≤3 years, respectively (P < 0.05), whereas no significant difference in 5-year overall survival (OS) was observed between the two groups (P > 0.05). In the multivariate analysis, treatment duration was not significantly associated with survival benefit (HR = 0.53, 95% CI 0.15–1.83, P = 0.313).

**Conclusion:**

Based on this retrospective study, adjuvant IM for more than 3 years did not independently improve prognosis in patients with high-risk GIST following radical resection. Moreover, the clinical significance of IM Cmin remains unclear. Therefore, extending adjuvant therapy beyond 3 years is not routinely recommended at present. These findings require further validation through prospective studies.

## Introduction

Gastrointestinal stromal tumors (GISTs) are the most common mesenchymal tumor of the gastrointestinal tract. They can occur in any part of the digestive tract, but most commonly in the stomach and small intestine (1). The median age of onset is 60–65 years old, and the annual incidence rate is about 10 cases per million population (2–4). GISTs are predominantly treated surgically, and about 60% of GISTs can be cured by surgery, but they are prone to recurrence and metastasis (5). Most GISTs with high-risk features recur within 5 years after surgery and are the main cause of death (6). Imatinib mesylate (IM), a highly selective tyrosine kinase inhibitor (TKI), has revolutionized the treatment paradigm for advanced gastrointestinal stromal tumors (GISTs), marking a pivotal milestone in the field of targeted therapy. As the established first-line agent for recurrent, metastatic, or unresectable GIST, IM also stands as the sole drug recommended by both domestic and international guidelines for adjuvant therapy following surgical resection (7, 8). However, the optimal duration of postoperative IM adjuvant therapy in patients at high risk with GIST remains controversial. The Z9001 study was the first to investigate the efficacy of postoperative IM adjuvant therapy. This study found that postoperative IM adjuvant therapy for 1 year significantly improved the disease-free survival of patients with medium and high risk of recurrence (9). Kang et al. (10) found that postoperative IM adjuvant therapy for 2 years is safe and can prolong the relapse-free survival of patients with a high risk of recurrence after complete resection. The SGXVIII/AIO study found that the efficacy of IM adjuvant therapy for 3 years was better than that for 1 year, and the risk of death was reduced by 50% after 10 years of follow-up (11). This study aims to analyze the relationship between the duration of IM therapy and postoperative prognosis in patients with high-risk GIST, thereby providing preliminary evidence for future research and clinical practice. Additionally, based on a limited sample, this study offers a descriptive analysis of plasma drug concentration data, intended to provide exploratory data support for subsequent large-scale investigations.

## Materials and methods

### Patient eligibility

This retrospective study was conducted at the First Affiliated Hospital of Chongqing Medical University, China. Only patients diagnosed with primary GIST between January 2008 and December 2021 were included in this study. The inclusion criteria were as follows: (1) Adult patients (aged ≥18 years); (2) pathologically confirmed GIST, with high-risk features for recurrence according to Guidelines of Chinese Society of Clinical Oncology for Gastrointestinal Stromal Tumors (2024 edition), defined by at least one of the following: longest tumor diameter > 10.0 cm; mitotic count > 10 per 5 mm^2^; a tumor diameter > 5.0 cm and mitotic count > 5 per 5 mm^2^; a small intestinal tumor diameter > 5.0 cm or mitotic count > 5 per 5 mm^2^; tumor rupture before or during surgery; (3) patients with GIST taking IM at a fixed 400 mg daily dose; and (4) good compliance (take IM regularly). The exclusion criteria were as follows: (1) serious comorbidities; (2) restricted oral administration because of significant gastrointestinal bleeding or obstruction; (3) presence of platelet-derived growth factor receptor alpha (*PDGFRA*) D842V mutation; and (4) IM adjuvant therapy not initiated within 8 weeks after radical surgery.

### Sample collection and pharmacokinetic analysis

To quantify IM plasma concentration, blood samples were collected 24 ± 3 h after the preceding dose in patients with GIST. Methods for blood sample processing, storage, and IM quantitative analysis have been described previously (12). The lower limit of quantification was set at 50 ng/ml.

### Data collection

Patients were routinely followed up by the GIST administrator at intervals of 3– 6 months. The clinical characteristics of all patients with GIST were documented, including age, sex, primary tumor site, maximum tumor diameter, mitotic count, tumor rupture, and gene mutation status. The primary endpoint was recurrence-free survival (RFS), defined as the time from the date of surgical resection to the first documented recurrence of GIST, confirmed either by histopathological or cytological examination, or by radiological assessment. Overall survival (OS) was defined as the interval from the date of surgery to death from any cause. Follow-up was conducted until March 11, 2025, or the date of patient death, whichever occurred first. Among the 75 patients, 48 (64.0%) were successfully followed up with complete adverse event data, while the remaining 27 (36.0%) were excluded from the adverse reaction analysis due to data limitations inherent in a retrospective study, such as incomplete medical records or loss to follow-up. The study protocol was approved by the Institutional Review Board and Ethics Committee of the First Affiliated Hospital of Chongqing Medical University (Ethical Approval No. 2025-737-01).

### Statistical analysis

All statistical analyses were performed using SPSS version 27.0. Continuous variables were compared using the Student’s t-test, while categorical variables were analyzed using the χ² test. Survival rates were estimated using Kaplan–Meier analysis, and between-group differences in survival curves were assessed using the log-rank test. Multivariable prognostic analysis was conducted using Cox proportional hazard regression models. Statistical significance was set at P < 0.05 <.

## Results

### Patient characteristics

**Figure 1:**
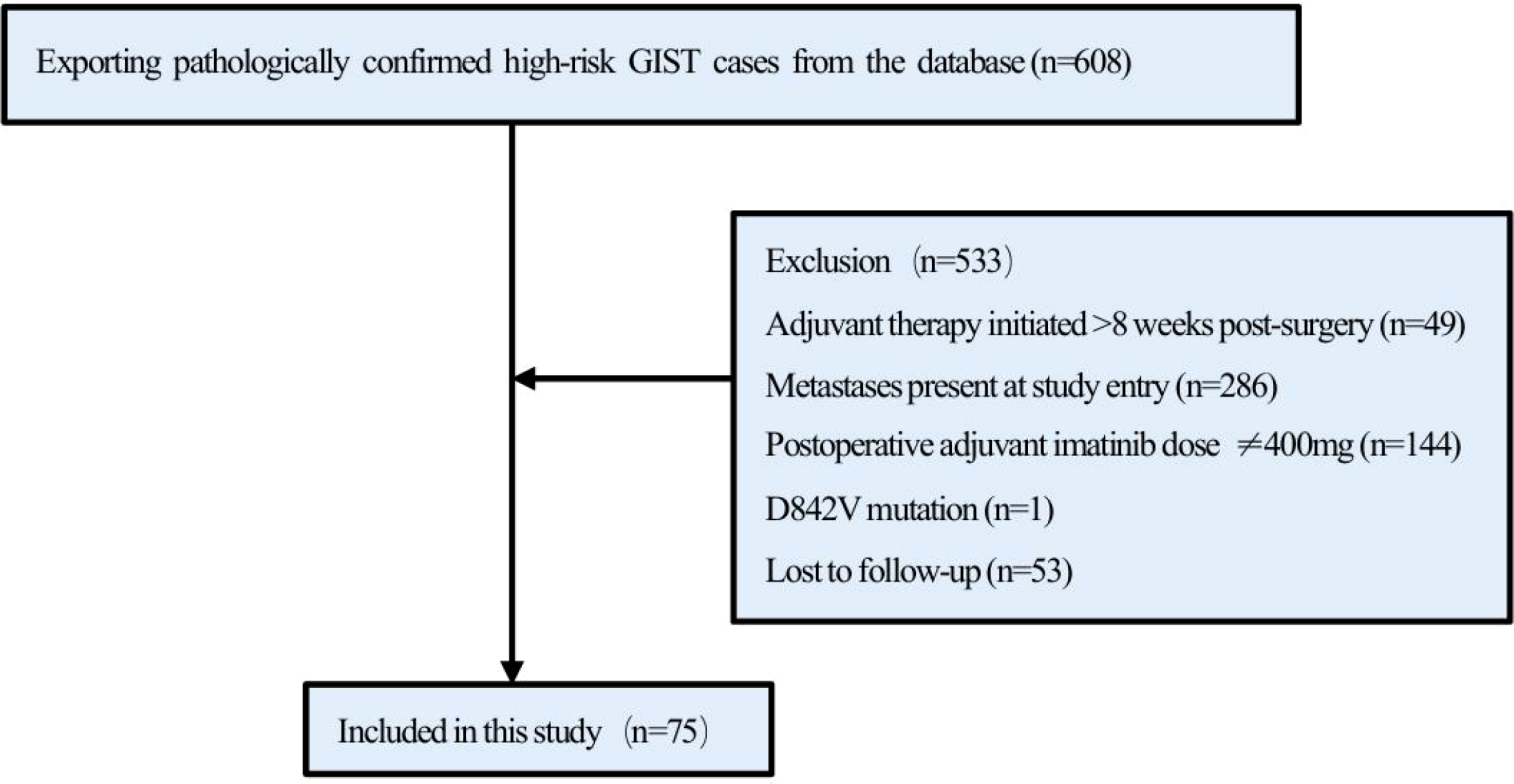
Flow chart of patient selection.

In total, 608 patients at high risk with GISTs were initially identified from the database. After excluding 533 patients for reasons such as not receiving postoperative adjuvant therapy with IM, non-radical resection, loss to follow-up, or other factors, 75 patients were ultimately included in the study (**Figure 1**). The median age of the patients was 53 years (range: 24–79), with a male predominance (56%; male-to-female ratio: 1.3:1). The most common tumor site was the small intestine (59%, n = 44), followed by the stomach (31%, n = 23). Furthermore, genetic testing was performed in 50 patients (67%). Among these, KIT proto-oncogene (*KIT)* exon 11 mutations were detected in 40 patients (80%), *KIT* exon 9 mutations in 4 patients (8%), and no *KIT* or *PDGFRA* mutations were identified in 3 patients (6%). The clinical characteristics of the patients are summarized in **Table 1**.

**Table 1:** The clinical features of the patients enrolled in the study.

Characteristics	No. (%)	
	≤ 3-year group (n=50)	>3-year group (n=25)
Age, median (range), y	56(24-79)	50(28-76)
≤65	38(76)	22(88)
>65	12(24)	3(12)
Sex		
Women	25(50)	8(32)
Men	25(50)	17(68)
Primary tumor site		
Stomach	15(30)	8(32)
Small intestine	31(62)	13(52)
Colon or rectum	3(6)	2(8)
Other	1(2)	2(8)
Primary tumor diameter, median (range), cm	7(1.6-17)	9(3.5-21)
<5.1	11(22)	7(28)
5.1-10.0	28(56)	8(32)
>10	11(22)	10(40)
Primary tumor mitotic count: local, median (range)	5(0-36)	5(0-25)
<6/5 mm^2^	34(68)	16(64)
6-10/5mm^2^	9(18)	5(20)
>10/5mm^2^	3(6)	2(8)
Not available	4(8)	2(8)
Tumor rupture prior to or at surgery		
No	40(80)	18(72)
Yes	10(20)	7(28)
Tumor mutation typed		
*KIT* exon 9	4(8)	–
*KIT* exon 11	24(48)	16(64)
Other	1(2)	2(8)
Wild type for *KIT* and *PDGFRA*	1(2)	2(8)
Not available	20(40)	5(20)

Percentages may not equal 100% because of rounding.

### Prognostic analysis

**Figure 2:**
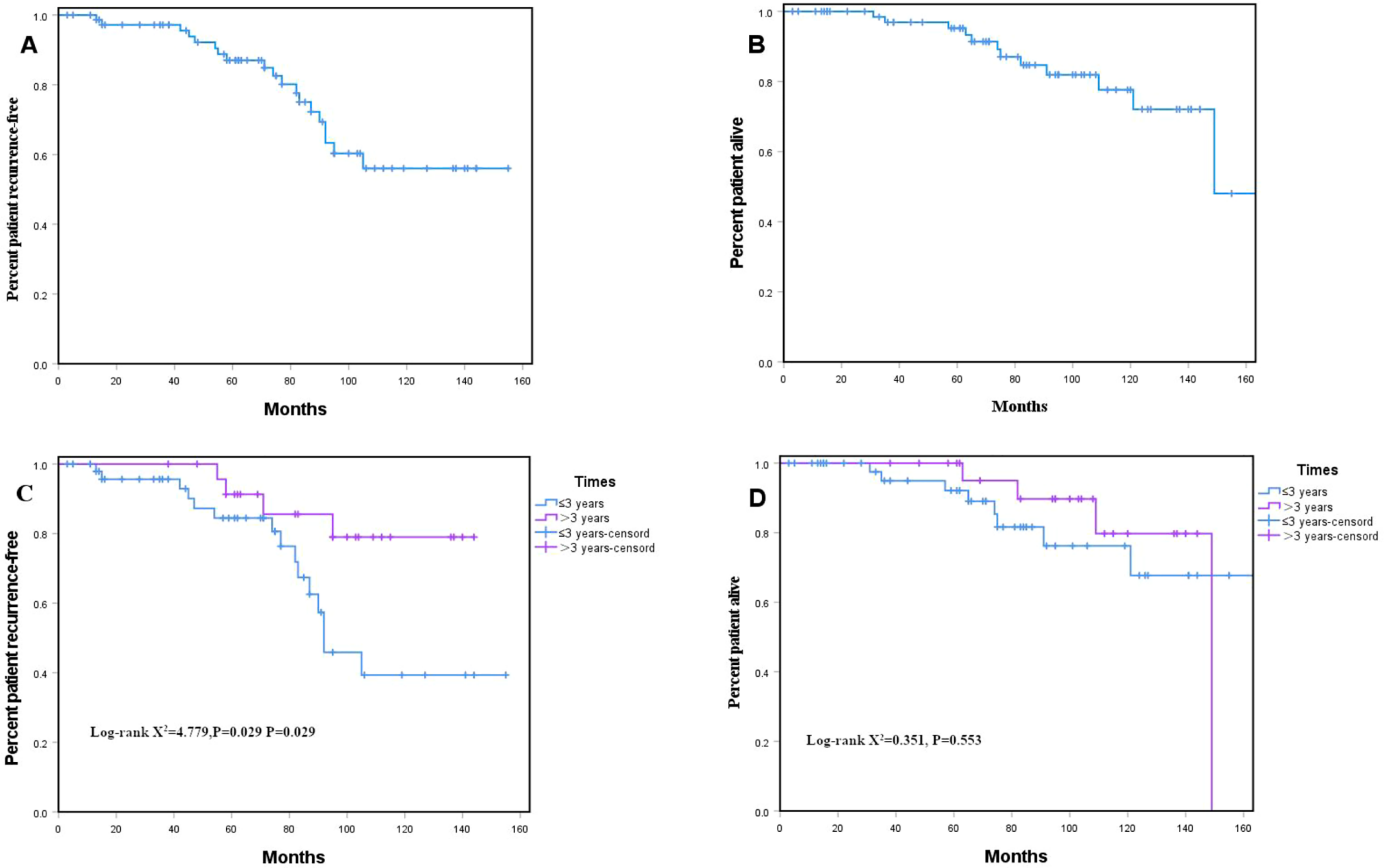
The recurrence-free survival (RFS) curve **(A)** and the overall survival (OS) curve **(B)** in the high-risk patients treated by imatinib; The RFS curve **(C)** and the OS curve **(D)** according to different durations of IM.

Among the 75 patients analyzed, the median follow-up duration was 77 months (range: 3–202 months). The 5-year RFS and OS rates for the entire cohort were 76% and 84%, respectively (**Figures 2A, B**). Univariate analysis showed that patients in the >3 years treatment group had a higher 5-year RFS rate than those in the ≤3 years group (84% vs. 74%, log-rank P < 0.05; **Figure 2C**). However, no significant difference was observed in 5-year OS between the two groups (both 84%, P > 0.05; **Figure 2D**). After adjusting for other covariates in multivariate Cox regression analysis, a treatment duration >3 years was not identified as an independent prognostic factor compared with ≤3 years (HR = 0.53, 95% CI 0.15–1.83, P = 0.313; **Table 2**). Univariate analysis showed that patients aged > 65 years had a 4.44-fold higher risk of death compared to those aged ≤ 65 years (95% CI: 1.38–14.27), and this difference was statistically significant (P = 0.012). Multivariate analysis further suggested that age > 65 years may be an independent adverse prognostic factor (HR = 7.24, 95%CI: 1.67–31.40, P = 0.008). In contrast, other clinicopathological factors, such as tumor location, size, rupture, and mitotic count, all exhibited P values > 0.05, indicating that their association with prognosis was not statistically significant(**Table 2**).

**Table 2:** Univariable and multivariable survival analysis for patients at high risk.

Variable	Univariate analysis			Multivariate analysis			
	N	HR (95% CI)	p-value	N	HR (95% CI)		p-value
Sex							
Male	42	–		43	–		
Female Age	33	0.969 (0.307-3.058)	0.957	33	–		–
≤65 years	60	–		60	–		
> 65 years Tumor sites	15	4.44( 1.38-14.27)	**0.012**	15	7.24	(1.67-31.40)	**0.008**
Stomach	23	–		23	–		
Non-stomach Tumor diameter	52	0.80( 0.24-2.65)	0.709	52	1.06	(0.28-3.99)	0.932
< 5. 1 cm	18	–		18	–		
≥5. 1 cm Mitotic count	57	1.05( 0.28-3.88)	0.944	57	0.49	(0.10-2.43)	0.381
< 6/5 mm2	56	–		56	–		
≥6/5 mm2 Tumer rupture	19	1.34( 0.35-5. 11)	0.668	19	2.10	(0.45-9.83)	0.344
Yes	17	–		17	–		
No	58	1.85( 0.40- 8.62)	0.436	58	1.39	(0.28-6.98)	0.691
Time of IM treatment							
≤3 year	50	–		50	–		
> 3 years	25	0.70( 0.21-2.32)	0.555	25	0.53	(0.15-1.83)	0.313

CI, Confidence Interval; HR, Hazard Ratio.

95% CI, 95% Confidence Interval. Bold value is statistically significant (P<0.05).

### Adverse reactions

In this study, a total of 48 patients were followed up, and their common adverse reactions were analyzed. (**Table 3**).

**Table 3:** Most frequently recorded adverse events.

Events	No. (%)		
	≤ 3-year group	>3-year group	P
	(n=28)	(n=20)	Value^a^
Any event	24(86.0)	12(60.0)	0.088
Hematological			
Anemia	3(10.7)	4(20.0)	0.429
Leukopenia	9(32.1)	6(30.0)	1.000
Thrombocytopenia	4(14.3)	1(5.0)	0.385
Nonhematological			
Periorbital edema	11(39.3)	8(40.0)	1.000
Leg edema	1(35.7)	1(5.0)	1.000
fatigue	8(28.6)	0	0.014
Nausea	9(32.1)	4(20.0)	0.512
Diarrhea	4(14.3)	6(30.0)	0.282
Loss of appetite	6(21.4)	4(20.0)	1.000
Rash	5(17.9)	0	0.066

^a^Fisher exact test.

According to the National Cancer Institute Common Terminology Criteria for Adverse Events, the most frequently observed adverse reactions included periorbital edema, leukopenia, nausea, and vomiting, with the majority of cases classified as Grade I or II. Grade III or IV adverse drug reactions were observed in 5 cases (18.5%) in the ≤ 3-year group and 2 cases (13.3%) in the > 3-year group. Treatment discontinuation due to adverse events was uncommon in the ≤ 3-year group, occurring in only two patients: one due to cerebral infarction and the other due to acute heart failure. In the > 3-year follow-up group, one patient discontinued treatment following a diagnosis of intrahepatic cholangiocarcinoma.

### Correlation analysis of IM plasma concentration

**Figure 3:**
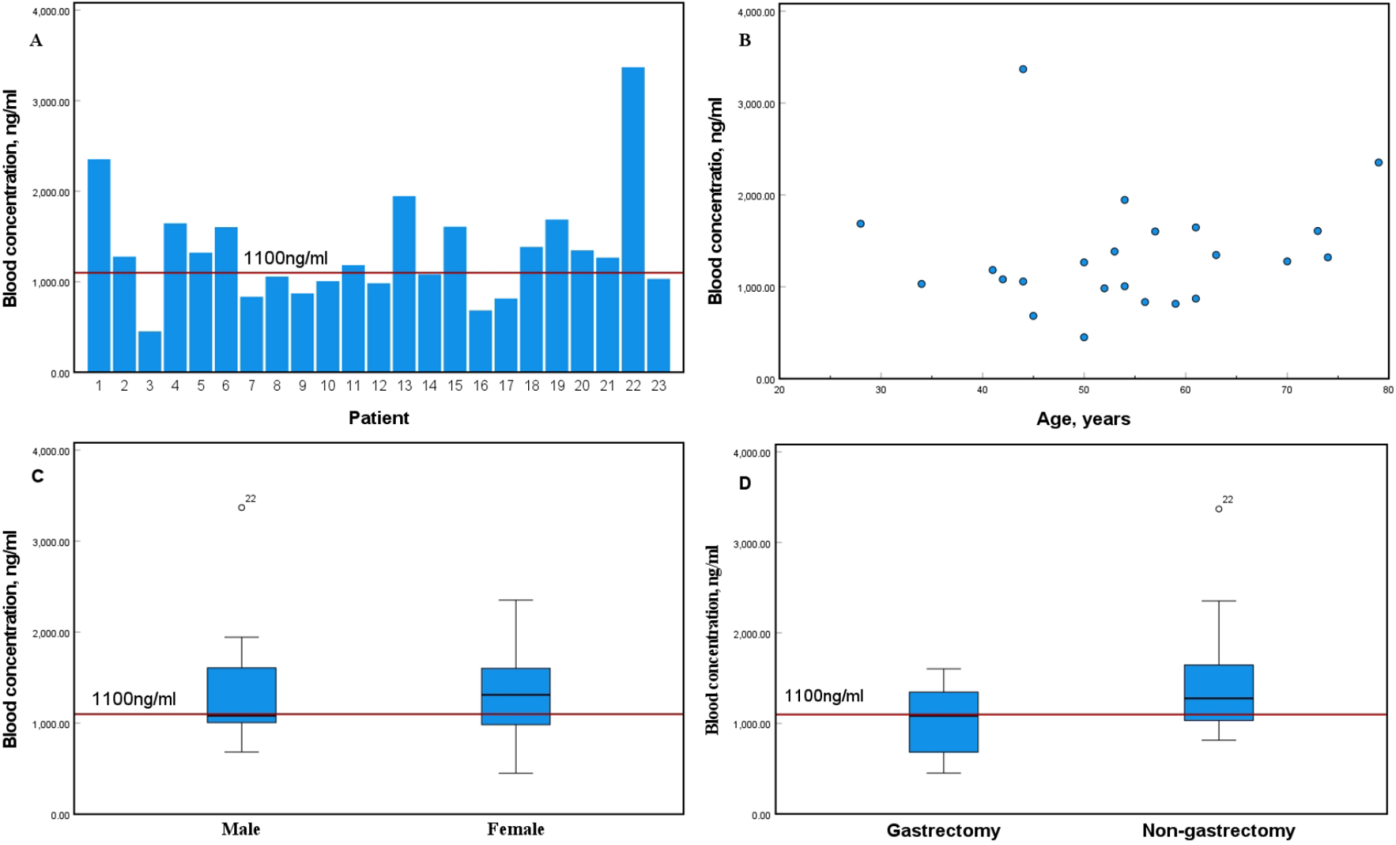
**(A)** Distribution Profile of Plasma Drug Concentrations; **(B)** Relationship Between Age and Blood Drug Concentration; Sex-blood drug concentration correlation; **(D)** Correlation between operative site and plasma drug concentration.

In this study, trough plasma concentration (Cmin) of IM was monitored in 23 patients (30.7%; **Figure 3A**), with 13 male (56.5%) and 10 female (43.5%) patients. Gastrectomy was performed in 6 patients (26. 1%) and not performed in 17 (73.9%). The cohort was stratified into two groups based on treatment duration: a ≤ 3-year group comprising 12 patients (52.2%) and a > 3-year group consisting of 11 patients (47.8%). The median IM Cmin was 1058.26 ng/mL (range: 451.17–2353. 17 ng/mL) in the ≤ 3-year group and 1271.83 ng/mL (range: 684.06–3369.52 ng/mL) in the > 3-year group.

Correlation analysis revealed no significant association between the IM blood concentration and patient age (r = 0.118, P = 0.592; **Figure 3B**). Furthermore, no statistically significant differences were observed in blood IM concentrations with respect to sex (t = 0.187, P = 0.846) or surgical approach (t = 1.407, P = 0. 174) as illustrated in **Figures 3C, D**, respectively. The 23 patients were stratified into three groups based on their Cmin levels: low (<760 ng/mL, n=2), medium (760–1100 ng/mL, n=8), and high (>1100 ng/mL, n=13). The corresponding mean ± standard deviation Cmin values were 567.6 ± 164.7 ng/mL, 960.5 ± 104.7 ng/mL, and 1691.8 ± 598.3 ng/mL, respectively.

## Discussion

GIST is the most prevalent form of sarcoma and serves as a paradigm for precision medicine in solid tumors (13). For advanced GIST, the tyrosine kinase inhibitor IM is the standard first-line treatment, while serving as an adjuvant therapy for tumors harboring *KIT* or *PDGFRA* mutations (14, 15). The ACOSOG Z9001 trial (9) demonstrated that compared with the placebo group, patients who received 1 year of adjuvant IM therapy had a significant reduction in the risk of postoperative recurrence by approximately 65% at 1 year. This study established IM as the standard adjuvant treatment for patient at intermediate to high risk of GIST recurrence after surgery, thereby changing the clinical practice guidelines. The subsequent SSG XVIII/AIO trial confirmed that postoperative adjuvant IM therapy for ≥ 3 years is the standard of care for these patients (16). The latest IMADGIST study (17) indicated that for patients with GIST with a recurrence risk > 35%, continuous IM treatment for 6 years resulted in a significant disease-free survival benefit (HR = 0.40) compared with the standard 3-year therapy. The magnitude of this improvement was comparable to the significant benefit achieved with 3 years of IM treatment versus 1 year in the SSG XVIII trial (HR = 0.6) (18). In this study, univariate analysis suggested that a longer duration of adjuvant IM therapy was associated with improved RFS (P < 0.05; **Figure 2C**), whereas no statistically significant difference was observed in overall survival (OS) (P > 0.05; **Figure 2D**), which may be attributable to the limited sample size. These findings indicate a potential link between extended adjuvant IM therapy and better RFS, suggesting a time-dependent treatment benefit; however, this observation should be interpreted with caution. Patients receiving treatment for more than 3 years may exhibit systematic differences in baseline characteristics or prognostic factors—such as better treatment adherence, higher socioeconomic status, improved treatment tolerance, or a tendency for clinicians to prolong therapy in those with a more favorable prognosis. These differences inevitably introduce selection bias, meaning that the observed improvement in RFS cannot be entirely attributed to longer treatment duration, but rather likely results from the interplay of multiple confounding factors. More importantly, although univariate analysis demonstrated a correlation between longer treatment duration and superior RFS, multivariate analysis failed to establish treatment duration as an independent predictor of outcome (HR = 0.53, 95% CI 0.15–1.83; **Table 2**). The wide confidence interval suggests insufficient precision in effect estimation, which may also stem from the relatively small sample size. Therefore, the RFS difference observed in univariate analysis warrants cautious interpretation. The current data do not support the conclusion that “a longer duration of adjuvant therapy independently confers better outcomes.” Future research should further investigate the relationship between treatment duration and clinical outcomes in larger prospective cohorts.

Tumor size and mitotic count are the most commonly used risk stratification parameters for prognostic assessment of GISTs. To investigate the pathological factors that have additional prognostic value in patients at high risk receiving standard adjuvant therapy, this study exclusively included patients undergoing adjuvant IM therapy. Univariate analysis revealed that age > 65 years was associated with an increased risk of patient death (P < 0.05; **Table 2**). Although this difference reached statistical significance, caution is warranted when interpreting the finding. Multivariate analysis further identified age > 65 years as an independent risk factor for patient mortality (HR = 7.2, 95% CI: 1.67–31.40; **Table 2**), suggesting a strong association between advanced age and elevated risk of death. However, given the relatively small sample size and the wide confidence interval of the hazard ratio, this result is subject to considerable statistical uncertainty. Therefore, the findings should be considered exploratory and require validation through larger prospective studies. Additionally, tumor location, tumor size, presence of rupture, and mitotic count did not demonstrate independent prognostic value in this study (P > 0.05; **Table 2**).

IM exhibits high oral bioavailability, with approximately 95% binding to albumin and α_1_-acid glycoprotein in human plasma. Its absorption, distribution, and metabolism are affected by physiological, pathological, genetic, demographic, and environmental factors (19). Female patients achieve a higher IM Cmin than male patients, which is often attributed to factors such as body weight and medication adherence (20, 21). Consistent with these findings, the mean IM plasma concentration in female patients in this study (2744.30 ng/ml) was relatively higher than that in male patients (2335.49 ng/ml), although the difference was not statistically significant (P > 0.05). In a randomized Phase II trial of IM (Study B2222), Demetri et al. reported that patients with advanced metastatic or unresectable GIST or both who achieved a Cmin ≥ 1100 ng/mL demonstrated significantly longer progression-free survival (PFS) compared to those with Cmin < 1100 ng/mL (22). Subsequent real-world data further confirmed that patients with Cmin ≥ 760 ng/mL achieved a longer PFS (23). The 23 patients were divided into three groups based on their trough concentration levels: low-concentration group (<760 ng/mL, n=2), medium-concentration group (760–1100 ng/mL, n=8), and high-concentration group (>1100 ng/mL, n=13). The mean trough concentrations ± standard deviations for each group were 567.6 ± 164.7 ng/mL, 960.5 ± 104.7 ng/mL, and 1691.8 ± 598.3 ng/mL, respectively. Given the limited sample size in the pharmacokinetic analysis of this study, no statistically powered inferences were drawn. Therefore, the findings should be interpreted with caution and serve solely as a basis for hypothesis generation in future research. Consequently, the value of therapeutic drug monitoring (TDM) in assessing the prognosis of patients with gastrointestinal stromal tumors warrants further validation in larger prospective cohorts. Although TDM may hold potential for individualized dose adjustment and prognostic evaluation in specific clinical scenarios—such as poor treatment response, suspected drug–drug interactions, or adherence issues—its routine implementation is not currently recommended by clinical guidelines. At present, the application of TDM in these contexts remains largely confined to research settings or individualized patient management, and further accumulation of evidence-based data is needed to elucidate its role and clinical value in routine practice.

This study has the following limitations. First, due to the constraints of the retrospective database, key confounding factors—such as mutation status of KIT exons 11 and 9—were not collected. The limited sample size (only 75 cases) and the high rate of missing molecular data (up to 40%) precluded adequate multivariable adjustment, sensitivity analyses, or propensity score matching. To avoid model overfitting, an excessive number of adjustment variables were not included; therefore, all results are reported as associations rather than effects, and conclusions are confined to correlational interpretations. Second, as a retrospective, single-center study, the design is susceptible to selection bias and measurement bias. Moreover, the long study period may have compromised the completeness and accuracy of adverse event data. Third, treatment duration did not show independent prognostic significance in the multivariable analysis (p = 0.313). Given the limited sample size and number of events, this null finding should be interpreted with caution. Differences in prognosis between groups cannot rule out the influence of imbalanced genotype distributions. Fourth, due to limitations of the retrospective design (e.g., incomplete medical records, loss to follow-up), 27 patients (36.0%) were excluded from the adverse event analysis. Additionally, to avoid classification bias resulting from ambiguous documentation, only adverse events with clearly documented data were objectively described. Fifth, the analysis of plasma concentration was based on a limited sample size, resulting in insufficient statistical power. The findings serve only as a basis for hypothesis generation in future studies and currently do not support clinical therapeutic drug monitoring. In conclusion, these findings require rigorous validation in larger, multicenter prospective studies that incorporate comprehensive confounding factors and therapeutic drug monitoring.

## Conclusion

In summary, this retrospective study did not demonstrate an independent and robust association between adjuvant IM therapy exceeding3 years and improved prognosis in patients with high-risk GIST following radical resection. Furthermore, due to limitations in sample size and data variability, no definitive conclusions could be drawn regarding the relationship between plasma drug concentrations and treatment efficacy or safety. These exploratory findings warrant validation through prospective studies with larger sample sizes. Therefore, extending adjuvant therapy beyond three years should not be routinely recommended until higher-level evidence becomes available.

## Data Availability

The data analyzed in this study is subject to the following licenses/restrictions: Clinical data involves patient privacy. Requests to access these datasets should be directed to Jie Zhang 3478220891@qq.com.
